# Therapy options for those affected by a long lie after a fall: a scoping review

**DOI:** 10.1186/s12877-022-03258-2

**Published:** 2022-07-15

**Authors:** Jenny Kubitza, Margit Haas, Lena Keppeler, Bernd Reuschenbach

**Affiliations:** 1 Department of Psychosomatic Medicine and Psychotherapy, Professorship of Spiritual Care and Psychosomatic Health, Technical University of Munich, Kaulbachstraße 22a, 80539 Munich, Germany; 2 Department of Nursing Science I, University Trier, Max-Planck-Straße 6, 54296 Trier, Germany; 3 Department Nursing Science, University Hospital Augsburg, Stenglinstraße 2, 86156 Augsburg, Germany; 4 Department of Health and Care, University of Applied Sciences, Preysingstraße 95, 81667 Munich, Germany

**Keywords:** Fall, Long lie, Inability to get up, Lying in one position after fall, Helpless, Older people, Frailty, Treatment, Fall management

## Abstract

**Background:**

After a fall, more than half of older people living alone are unable to get up or get help independently. Fall-related recumbency makes affected individuals aware of functional status limitations and increased vulnerability. Patient-centered therapy is needed to manage physical, psychological, and social needs. This review summarizes the current evidence on care options for the special patient population.

**Methods:**

The scoping review used the six-step framework proposed by Arksey and O´Malley and was conducted in accordance with the modified *Preferred Reporting Items for Systematic Reviews and Meta-Analyses (PRISMA)* framework for scoping reviews. The literature searches were conducted in five databases and ten online archives. Articles were screened, assessed and selected using defined inclusion and exclusion criteria. Articles were included if they were published in either German or English and related to the care of long lies. Thematic synthesis was based on the literature review.

**Results:**

The search yielded 1047 hits, of which 19 research papers were included. Two themes were identified: (1) acute therapy, focused on prolonged recumbency and pronounced physical effects; and (2) preventive therapy, which examined standing up training, technical aids, and social control systems in the context of fall management.

**Conclusions:**

There are a limited number of interventions that relate to the patient population. The interventions are predominantly presented independently, so there is a lack of structuring of the interventions in the form of a treatment pathway. In addition to pooling professional expertise and an interprofessional approach, it is important to continue inpatient treatment in the home setting, even though the effectiveness of interventions in a home setting has hardly been verified thus far. The solution for a missing treatment process is first of all a planned, interprofessional and intersectoral approach in therapy.

**Supplementary Information:**

The online version contains supplementary material available at 10.1186/s12877-022-03258-2.

## Background

The number of falling accidents increases with age. In the age group of 65 years and older, every third person falls annually [1]. After an accident, older persons have difficulties to independently getting out of the situation [2–4]. Half of those over 65 years of age who fall are found on the floor by rescue services [2, 3]. Among persons in need of care, 70% of those who fell already had problems getting up [4]. Since most elderly individuals spend most of the day alone in their own homes, they have to wait until the alarm is raised and help arrives [5]. One in eight older individuals who fall report lying on the floor for more than an hour [2, 4]. The main reason for fall-related recumbency is rarely an injury. The majority of affected individuals present trivial fall-related lesions [3, 6]. Nonetheless, individuals who fall and have limitations related to standing experience increased hospitalization rates [3, 6]. Individuals with a long lie are primarily diagnosed with fluid deficit and electrolyte disturbance, hypothermia and infections such as pneumonia or urinary tract infection, and skin damage and pain [3, 6]. In the first three days after a long lie, declines in mobility and reduced activities of daily living are also observed in affected individuals [3, 6]. Due to the sudden and unwanted state of reduced mobility, affected individuals become aware of their own physical, psychological, and social limitations in a state of need [7]. This results in decreased self-esteem, which increases the risk of permanent deterioration in a self-help status. Individuals affected by of long lies show losses in autonomy and increasingly withdraw from an active life [3, 6, 8]. In the first year after the incident, hospitalization and mortality rates increase in affected individuals [3, 6].

Long lies are an impactful experience for affected individuals and affect their well-being and quality of life both acutely and in the long term. Holistic interventions require interprofessional and cross-setting approaches [7].

A preliminary search of MEDLINE, the Cochrane Database of Systematic Reviews was conducted and no current or underway reviews on the topic were identified. Several reviews addressed the prevention of falls in older people and treatment after a fall but have missed the long lie after the fall.

The aim of this review is to provide an overview of studies that address the prevention and treatment of long lies. The following research question was identified:

1. How is a holistic and interdisciplinary therapy provided to those affected by a long lie after a fall?

## Methods

As the research question is a complex issue, the first step should be to get an orientation in the state of research. The research topic should be analyzed broadly, and the research gaps should be identified. The scoping review followed the six steps proposed by Arksey and O´Malley Framework: (1) identifying the research question, (2) identifying relevant studies, (3) study selection, (4) charting the data, (5) collating, summarizing and reporting the results, and (6) optional step: consultation exercise [9, 10].

Inclusion and exclusion criteria for the reviewed studies: Screening of the results lists was performed with specified inclusion and exclusion criteria oriented to the PCC-framework (Population, Concept, Context) [9, 10]. Included were studies that addressed the aspects of *care of long lies by health care professionals, outpatient and/or inpatient settings, and prevention of long lies.* In view of the low number of hits, all study designs were included without temporal restrictions, provided they were scientific publications in German or English as well as guidelines or standards. Studies focusing on the care of falls without long lies were excluded, as the consequences of falls with recumbency differ from the effects of a simple fall event.

Search strategy: 3 main components were used in the search process; long lie, fall, and treatment were expanded with truncations and linked by the Boolean operators AND and OR. Table 1 provides an overview of the total search terms.

**Table 1:** Key concepts and search terms

	**Population**	**Concept**	**Context**
Search terms and synonyms	long lie* OR inability to get up OR immobil* OR helpless OR rhabdomyolys* OR trauma fall* OR drop*	therap* OR treatment OR nurs* OR care*	As all settings, no keywords
MeSH Terms PubMed	Falls, Accidental falls	Therapy, nursing, rehabilitation	-

First a preliminary search was pilot tested to pre-select key words from abstracts and titles of papers considered relevant to the topic. This step only involved the database PubMed. The key words from abstracts and titles of relevant papers and Mesh terms were used to define and develop a search strategy (see Additional file I). The search strategy was applied to all databases and adapted as needed. In addition to the PubMed, CINAHL, Cochrane, GeroLit, and LIVIVO databases, the online archives of 3 German journals were searched (Pflege, Pflegewissenschaft, Pflegezeitschrift). Searches for German- and English-language standards and guidelines were conducted in the archives AHRQ, NICE, DQNP, ZQP, NVL, AWMF, and IQWIG. Citation tracking from full-text articles supplemented the identification of available literature. The systematic search was conducted from September 2019 to July 2020 and in August 2021, the literature search was updated to include new publications since the original search. The search conducted in August 2021 was extended to include free searches on Google Scholar.

Study selection: Study screening and selection were guided by the modified *PRISMA statement* for scoping reviews [11]. After removal of duplicates, a preselection was made using the titles and abstracts. The full texts of the remaining publications were reviewed for inclusion criteria. Disputed studies were assessed by 2 authors and subsequently compared. Critical appraisal: To get an overview of the quality of studies on the research topic, an assessment of study quality was performed with the help of the Standard Quality Assessment Criteria. The qualitative and quantitative studies were evaluated with different criteria. The assessment of the quantitative studies has a total of 14 criteria. Because 2 criteria did not apply to any of the 11 quantitative studies, the studies were assessed with 12 criteria. The qualitative studies were evaluated with 10 criteria. No criteria were allowed to be removed here. The criteria were scored as Yes (2 points), Partial (1 point), and No (0 points) [12]. As this is a scoping review, no study was excluded on quality grounds.

Data extraction and synthesis: The extraction and synthesis of findings from the selected studies was guided by the thematic analysis of a *literature review* [13]. The first author screened the studies several times and filtered information on design, setting, sampling, intervention and key outcomes. A data extraction sheet was used to reduce the features to a manageable set and to point out their credibility and limitations. The data were then compared and sorted thematically. The themes were reviewed by additional authors. The synthesis is presented in narrative form.

## Results

### Search outcomes

**Fig. 1:**
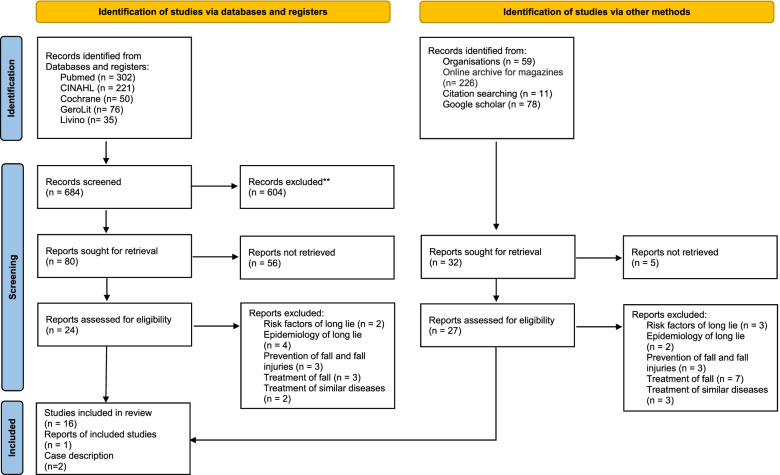
Flow chart of search and screening process

An initial keyword search yielded 1047 articles. Using citation tracking, 11 additional publications were added to the search. Duplicates were removed and the remaining titles and abstracts were screened against the defined inclusion and exclusion criteria, leaving 51 articles for full-text review. The reasons for study exclusion and the results are explained in Fig. 1 (PRISMA flow diagram). 19 publications were included in the synthesis.

### Study description

A total of 1276 subjects from 5 countries were included in the 19 studies, with one experimental laboratory study not reporting subject numbers [14]. The studies included (a) individuals affected by long lies [15–18], (b) individuals who fell or were at risk of falling with limitations in their ability to get up from the floor [19–25], (c) providers of patients who experienced long lies [26, 27], and participants with characteristics (b) and (c) [28]. 5 studies focused on (d) individuals with and without functional limitations; these were experimental laboratory studies [14, 29, 30] and comparative cross-sectional studies [31, 32]. The sample sizes ranged from 1 to 367 participants. Data collection occurred from 1993 to 2019. The majority of studies were based on a quantitative design. In addition to 4 experimental studies [14, 19, 29, 30] and 5 cross-sectional studies [18, 21, 27, 31, 32], 2 retrospective studies [23, 24] and 1 prospective study [22] were conducted. 3 studies had a qualitative design [25, 26, 28], and the review was a qualitative meta-synthesis [20]. In addition, 2 case descriptions [15, 16] and 1 single case study [17] were included. 3 publications described acute therapy [15, 16, 26]. The remaining 16 studies focused on tertiary prevention interventions [17, 19] and secondary prevention interventions in the context of fall management [14, 18, 20–25, 27–32]. Fall management refers to counseling [20, 27, 28], standing up training [17, 19, 21, 31, 32], technical aids [14, 22–25, 29, 30] and social control systems [18]. Further details on the characteristics of the 19 studies are provided in Table 2.

**Table 2:** Characteristics of the studies included in systematic review

Author & year	Country of Origin	Focus of research	Methods	Sample and Sample Size	Type of therapy
Häcker & Offterdinger 2019	GER	Describing interventions in acute care settings	Case description	72-year-old subject with long lie ≥ 5d	Primary care in home setting
Hierholzer et al. 2013	GER	Describe the diagnostic process and interventions in acute care settings	Case description	Older subject with long lie ≥ 24 h	Primary care in home setting
Fischer 2019	CH	Explaining diagnostic procedures and interventions in acute care	Qualitative descriptive study with expert interviews	Nursing and medical professionals in the field of emergency care *n* = 4	Primary care in the emergency department
Reece & Simpson 1996	GB	To record the learning outcomes of standing up training comparing forward-chaining and backward-chaining approaches	Descriptive Experimental Study	Older persons who had fallen and were unable to get up from the floor *n* = 38	Physiotherapy in rehabilitation facility
Adams & Tyson 2000	GB	Capturing the effects of standing up training using the backward-chaining approach on mobility	Single case study	79-year-old female subject with long lie ≥ 12 h	Physiotherapy at home
Simpson & Salkin 1993	GB	Acquisition of content and implementation of fall management	Cross-sectional study	Physical and occupational therapists *n* = 67	Prevention in the form of fall management in inpatient and outpatient settings
Charlton et al. 2017	AUS	Explaining content and implementation of fall management	Qualitative Meta-Synthesis	≥ 65-Year-old persons at risk for falls with limitations in ability to get up from the floor *n* = 112	Prevention in the form of fall management for the home
Charlton et al. 2016	AUS	Explaining factors influencing planning for fall management	Qualitative study using semistructured interviews and focus group	Therapeutic staff *n* = 7 ≥ 65-Year-old persons at risk for falls with limitations in ability to get up from the floor *n* = 7	Prevention in the form of fall management for the home
Schwickert et al. 2016	GER	Capturing the ability to stand up in comparison between age groups	Cross-sectional study	Persons between 20 and 50 years *n* = 14 Persons ≥ 60 years *n* = 10	Prevention in the form of fall management for the home
Alexander et al. 1997	USA	Capture of ability to stand up compared between age groups and degree of mobility limitations	Cross-sectional study	Persons of a younger age *n* = 24 Older persons without limitations *n* = 42 Older persons with limitations *n* = 38	Prevention in the form of fall management for the home
Ardali et al. 2019	USA	Testing the reliability and validity of the Floor Transfer Test as a measurement tool for assessing physical markers	Cross-sectional study	≥ 65-Year-old persons at risk for falls *n* = 61	Prevention in the form of a home assessment
Gurley et al. 1996	GB	Capturing social networks	Cross-sectional study	Older fallen persons with lying time ≥ 1 to 72 h *n* = 367	Prevention in the form of fall management for the home
Fleming & Brayne 2008	GB	Capturing reasons for non-usage of emergency call buttons	Prospective cohort study	≥ 90-year-olds who fell and were unable to get up from the floor *n* = 110	Prevention in the form of fall management in outpatient and long-term inpatient settings
Heinbüchner et al. 2010	GER	Capturing reasons for non-usage of emergency call buttons	Retrospective study with interviews	Older persons who fell with limitations in ability to get up from the floor *n* = 52	Prevention in the form of fall management for the home
Johnston et al. 2010a	AUS	Capturing reasons for non-usage of emergency call buttons	Retrospective study	≥ 65-Year-old persons with limitations in ability to get up from the floor *n* = 268	Prevention in the form of fall management for the home
Johnston et al. 2010b	AUS	Explanations for non-usage of emergency call buttons	Qualitative study using semistructured interviews	≥ 65-Year-old persons with limitations in ability to get up from the floor *n* = 31	Prevention in the form of fall management for the home
Aziz et al. 2007	CAN	Testing the specificity and sensitivity of sensor systems for the detection of fall-related lying times	Experimental laboratory study	Younger subjects *n* = 10	Prevention in the form of fall management for the home
Ariana et al. 2012	AUS	Testing of the specificity and sensitivity of motion detectors for the detection of lying times due to falls	Experimental laboratory study	Subjects in the age range of 45 to 87 years	Prevention in the form of fall management for the home
Bourke et al. 2008	CAN	Testing the specificity and sensitivity of sensor systems for the detection of fall-related lying times	Experimental laboratory study	Younger, male subjects *n* = 11	Prevention in the form of fall management for the home

### Critical appraisal

A summary of the critical appraisal is shown in Table 3.

**Table 3:** Assessment of the study quality

Qualitative studies		
Study	Total score /20 points	Total score /100%
Charlton et al. [20] (2017)	20	100%
Charlton et al. [28] (2016)	17	85%
Adams & Tyson. [17] (2000)	16	80%
Johnston et al. [25] (2010b)	13	65%
Fischer [26] (2019)	6	30%
Häcker & Offterdinger [15] (2019)	4	20%
Hierholzer et al. (2013) [16]	4	20%
**Quantitative studies**		
**Study**	**Total score /22 points**	**Total score /100%**
Aziz et al. [29] (2017)	22	100%
Schwickert et al. [31] (2016)	21	95%
Ardali et al. [21] (2019)	20	91%
Ariana et al. [14] (2012)	20	91%
Fleming & Brayne [22] (2008)	20	91%
Gurley et al. [18] (1996)	20	91%
Heinbüchner et al. [23] (2010)	20	91%
Reece & Simpson [19] (1996)	19	86%
Bourke et al. [30] (2008)	18	82%
Johnston et al. (2010a) [24]	18	82%
Alexander et al. [32] (1997)	17	77%
Simpson & Salkin [27] (1993)	12	55%

Two studies have reached a high level of evidence (90–100%). These were one quantitative study [29] and one qualitative study [20]. The evidence is lower with a mean score of 56% for the qualitative studies, compared to 89% for the quantitative studies. Three qualitative studies have low evidence (< 50%). These are the studies on acute therapy. They lacked transparency in data collection and analysis. The 2 case descriptions narratively described the initial therapy of long lies and did not specify the methodology [15, 16]. The third study (qualitative expert interviews) did not adequately present the data analysis, but intersubjective comprehensibility was enhanced by the attached transcribed data [26]. 14 studies have moderate evidence (50–90%). In the qualitative studies, the methodological limitations are that the sampling strategy was not adequately described [28], and the number of subjects was with 1 elderly person small [17]. The third qualitative study on the use of low technology lacked a transparent presentation of the data analysis, which is why limited credibility must be assumed [25]. Among the quantitative studies, 10 were rated moderate. The quantitative studies were also limited because of sampling. The studies excluded individuals who lived in multiperson households [18], persons with poor knee and hip flexibility [19], and 1 study excluded subjects who could not stand up six consecutive times [32]. This excludes factors from the studies that applied to the high-risk group and the better functional status of participants must be taken into account [19, 32]. The studies of fall management counseling were conducted with health care stakeholders. When collecting counseling content and needs, subjects were able to share their thoughts on the topic prior to data collection [27, 28]. Studies on technical aids must distinguish between high technology and low technology. Testing of the specificity and sensitivity of high technology took place exclusively in laboratories with younger subjects [14, 29, 30]. Low technology was used in the home of the test persons. Participants' self-assessments may have led to error reporting [22–24].

Despite the listed limitations, 15 studies were rated with high or moderate evidence. The studies were all surveys of the prevention of fall-related long lies [14, 17–25, 27–32]. The 3 studies on acute therapy for sufferers of long lie have only low evidence [15, 16, 26].

**Fig. 2:**
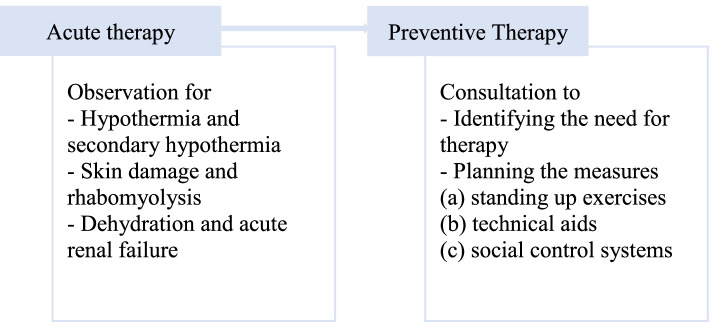
Main Themes

Findings: An overview of the key themes of the review is shown in Fig. 2.

### Theme 1 acute therapy

Initial care focuses on the physical stabilization of the affected person. When a person is found lying on the floor, it must first be assumed that the situation is acutely life-threatening or that there is potential for acute deterioration of the general condition [15, 16, 26]. Diagnoses associated with prolonged long lies include (1) hypothermia and secondary hypothermia, (2) skin damage and rhabdomyolysis and (3) dehydration and acute renal failure.

(1) Hypothermia and secondary hypothermia: Measurement of body temperature must always be performed on individuals lying on the ground [15, 16, 26]. In the event of mild hypothermia, victims should be protected from further cooling [16, 26]. For this purpose, it is recommended to close windows [15] and to offer the affected persons a head covering or a warm blanket [26]. In cases of pronounced hypothermia, it must be assumed that to protect the body core and vital organs, the blood vessels in the periphery have constricted and the extremities have cooled down [15, 26]. Consequently, rewarming measures should focus on the torso area and should be performed cautiously [15]. A warming blanket is a suitable option [26]. Warming massages are explicitly not recommended [15]. In addition to warming measures, mixing of the cold and overacidified blood from the extremities with the warm blood of the body core must be prevented [15, 16, 26]. Pronounced changes in position can be avoided by refraining from elevating the arms and legs [15] and transporting to the acute inpatient setting in a horizontal position under circulatory monitoring [16]. The use of a vacuum mattress is advised for transport [16]. When dealing with hypothermic victims, a cautious approach is critical to prevent cardiac arrhythmias and the resulting secondary hypothermia [15, 16].

(2) Skin damage and rhabdomyolysis: Individuals found on the ground must be examined for skin damage and pressure ulcers [16, 26]. Here, a special focus should be placed on the body regions contaminated by excreta [26]. Pressure ulcers and their severity provide initial indications of a long lie duration [16] and a possible increase in myoglobin and creatine kinase in the blood [16, 26]. If the pressure ulcers are pronounced, a long recumbency period and the risk of rhabdomyolysis should be assumed [16, 26].

(3) Dehydration and acute renal failure: An initial check of fluid balance can be integrated during the body check [26]. Signs of exsiccosis lead to observation of urine for quantity and color. A reddish-brown discoloration of the urine is indicative of myoglobinuria [26]. Initiation of infusion therapy is recommended to prevent acute renal failure [16, 26].

Initial care includes both ambulance service [15, 16] and hospital emergency departments [26]. When patients are transferred to the acute inpatient setting, their length of stay must be clearly communicated [16].

### Theme 2 preventive therapy

Preventive therapy includes (1) counseling, (2) standing up training, (3) technical aids and (4) social control systems as part of a falls management program.

(1) Counseling:

Effective planning of each aspect should be performed during counseling [20, 28]. Feelings of dependency [20] and the need for assistance [28] occur in older persons during the planning of fall management. It is the role of the counseling person to address fears and assist affected individuals in reducing their worries [20, 28]. Subsequently, it is recommended to gently guide individuals in recognizing their own fall risk [20, 25, 28] and to educate them about the need for fall management [28]. In further planning for fall management, multiple support services should be presented to affected individuals [20, 25, 28]. This allows for informed and free choice and promotes feelings of autonomy and control [25, 28]. However, health care professionals can make recommendations [28]. The integration of caregivers has a positive impact on fall management planning [28]. To empathically and effectively guide older persons in planning and implementing fall management, health care professionals need continuing education that informs them about critical interventions [25, 28].

(2) Standing up training:

Standing up training can be implemented as both a tertiary prevention [17, 19] and secondary prevention intervention [22, 26, 29, 32, 33] under the guidance of physical therapists. Older individuals are assisted in finding a movement sequence that suits them [26, 32, 33]. For learning movement sequences, standing up training in the form of the forward chaining method [19] and training in the form of the backward chaining approach [17, 19] are recommended. In both methods, the exercise is broken down into individual substeps that are learned sequentially. Both approaches result in improvements in the ability to get up from the floor [17, 19], but cooperation and consent rates are higher with backward chaining training [19]. The backward chaining approach allows individuals to go through the steps of standing up backward and ensuring that their abilities are sufficient to stand up [19]. Progress in training has a positive effect on the self-confidence and mobility of affected individuals and encourages their compliance [17]. When training success is low, it is recommended to focus on locomotion on the floor, with the goal of reaching aids placed on the floor, such as a phone or blanket [19].

In addition to learning movement sequences, regular participation in a muscle-building training program is part of standing up training [31]. The program should train the leg, arm, and abdominal muscles [31, 32] as well as the sense of balance [25, 28]. Strengthening of the upper and lower extremities has a positive effect on the ability of older persons to get up from the floor [31]. In cases of severe physical limitations, an assessment should be performed to evaluate mobility before standing up training [21, 28].

(3) Technical aids: Technical aids allow timely contact of assistive personnel and reduction of long lie times [14, 22, 25, 29, 30]. Both high-tech and low-tech assistive devices should be introduced in a consultation [20]. Wearable sensor systems [29, 30] and motion detectors [14] are listed as high-tech aids. Wearable sensor systems can distinguish falls from activities of daily living as well as near falls [29, 30]. Alternatively, home monitoring technology can be used. Motion detectors detect fall situations from which individuals cannot extricate themselves independently and contact assistive personnel [14]. It is recommended to place a motion detector in the upper and lower halves of a room. Motion detectors are most suitable for one-person households [14]. Although high-tech aids have the potential to prevent long lies [14, 29, 30], the majority of older persons make a conscious decision not to use high-tech aids. Reasons cited include fears regarding the financing and usage of the technologies as well as invasion of privacy [20]. Low technology, such as the home emergency call system or cell phone, is advised [20, 28]. Sufficient acceptance and understanding of the system have a positive effect on wearing [23–25] and activating the alarms [22–24]. Older persons' satisfaction with the system only slightly influences regular wearing of the home emergency call button; more decisive is the assessment of one's own fall risk as well as the need for the home emergency call button [20, 23, 24].

(4) Social control systems: Another component of fall management represents the establishment and active promotion of social connection [18, 25, 28]. With the help of the social network, the absence of a fallen person can be noticed promptly [18].

## Discussion

The amount of published literature on therapy for long lies is small. The specific research question and the inclusion and exclusion criteria reduced the number of hits to 19 publications. To avoid further reductions in content, publications that showed methodological opacity and were rated with low evidence were also included in the review. In total, 3 studies were rated with low evidence [15, 16, 26]. The 3 studies were classified under the topic area of acute therapy. Acute therapy was surveyed with the help of 2 older individuals who had suffered long lies [15, 16] and with 4 emergency care professionals [26]. The treated individuals were exposed to a long recumbency period of 24 h [16] and 5 days [15]. Length of long lie affects case descriptions of initial care, which primarily explain the management of potentially life-threatening situations [15, 16] and focus on sequelae caused by prolonged immobility [16, 16, 26]. However, only 15% of recumbent fallers show serious physical injuries [3]. The majority of them predominantly require assistance to stand up [24] and clarification for the impaired ability to stand up [3]. The initial treatment for fallen persons with a short recumbent duration as well as a low degree of injury remains unexplained in the 3 studies [15, 16, 26].

The study quality and quantity for the treatment of long lies are much lower than those for the prevention of long lies. Preventive measures mainly focus on the early identification of potentially vulnerable individuals and prevention of long lies through targeted interventions [14, 18, 20–25, 27–32]. The studies clearly show that recognition of one's own fall risk and acceptance of the measures are crucial for compliance with preventive therapy [20, 22, 24, 25, 27]. Counseling sessions addressing the need for fall management and the feelings of dependence and vulnerability it triggers are advised [20, 23–25, 28]. In this regard, 3 studies conducted a comparative analysis with older persons and persons at risk for falls to capture reasons for effective use of the home emergency call system [23–25]. The quantitative surveys were supplemented by interviews with persons at risk of falling [20, 23, 25, 28] and therapists [28]. Nevertheless, it must be critically reflected that the findings on the effective use of technical aids among older persons and persons at risk of falling are only partly up to date due to advances in technology toward more user-friendly systems [23–25]. However, because satisfaction with the systems has only a minor influence on regular use compared with the assessment of one's own fall risk, further development of technical aids can be set aside [20, 23, 24].

In addition to counseling and technical aids, social networks are listed as a preventive measure. One study explicitly examined the measure of social control systems [18]. Since the study was published in 1996, changes in social structures have to be considered [18]. Two qualitative studies in 2010 [25] and 2016 [28] confirmed the 1996 recommendations and added further social control systems interventions.

The very low number of hits on the therapy of long lies as well as on preventive therapy could probably have been increased by searching additional databases and by the MeSH term “prevention and control”. However, most studies investigated how to prevent long lies after a fall [14, 18, 20–25, 27–32] and none of the studies were published in a nursing science journal, although the databases had the appropriate focus. The studies were published in acute care [15, 16, 24], medical [18, 22, 25], geriatric [23, 27, 31, 32], therapeutic [17, 19, 21], rehabilitation [28], and multidisciplinary journals [14, 20, 29, 30].

Recommendations for future research: Research projects on fall management have reviewed interventions designed to prepare older persons for a fall situation. The extent to which the interventions can be applied independently in the individual's own home and the effectiveness of fall management in preventing long lies in the home setting of older individuals remains to be determined. Research on the treatment of patients who experience long lies is in its infancy. There are few studies examining the treatment of this special patient population. Differentiation between fallen individuals who experience long lies and those without long lies rarely occurs. Initial approaches can be seen in acute medical [15, 16, 26] and physiotherapeutic care [17, 19]. Studies with a higher number of subjects should be conducted to verify the findings [15–17, 19, 26]. The treatment of sufferers with a short recumbency period is not mentioned [15–17, 26]. It also remains to be seen to what extent trauma therapy as a psychological intervention influences the healing process. Long lies make sufferers aware of the limits of their ability to act and pose an extraordinary threat in the lives of individuals. Sufferers of long lies show signs of psychological trauma [3, 6–8]. Negative feelings such as threat, fear and helplessness are expressed after long lies [7] and are manifested by a permanent deterioration in self-help status [3, 6, 8].

Evidence of poorly differentiated treatment for those affected by long lies is reflected in the increased rates of hospitalization and mortality among this population [3, 6, 18]. In a one-year prospective cohort study, 60% of older fallers with at least one hour of a long lie were readmitted as a result of a fall. A total of 29% required transfer to an inpatient long-term care facility [22]. To maintain functional status and independence, the development and review of a treatment pathway for patients who experience long lies is a useful addition.

Implications for practice: The evidence from the studies indicates that therapy should focus on helping older persons to recognize their own risk of falling and the danger of lying down for a long period of time. Awareness is a precondition for older people to participate in falls management [20]. The results of acute therapy should be considered very critically because of the low evidence. Even though these are case descriptions of current date, it can only be assumed that the acute care as described corresponds to the current standards of the ambulance service and the acute care setting [15, 16, 26]. In addition to the somatic aspects, it is imperative to consider the psychological consequences of the long lie. Therefore, both individuals with long and short recumbency due to an inability to get up from the floor should be accompanied to the hospital to clarify the causes of long lies and initiate fall management.

## Conclusion

This study shows that there is rarely a distinction in therapy between persons who have fallen and have been able to get back up immediately and persons who have fallen with long lies. The therapy for a long lie is only slightly adapted to the needs of the affected persons. The limited number of studies on the care of long lies does not allow evidence-based conclusions to be made about the treatment pathway for older persons found on the ground. It is recommended that treatment be accompanied by multiple professionals and continue in the home setting. Of particular importance in this regard are the primary caregivers who accompany the affected persons to the acute inpatient setting, the nursing professionals who, in the context of a consultation, support the affected person in recognizing a need for treatment, and the physical therapists who can influence the patient's self-confidence by strengthening physical abilities. Psychological or psychotherapeutic services are required to counter the psychological consequences of the long period of helplessness and, in some cases, fear of death.

## Supplementary Information

**Additional file 1.**

## Data Availability

The datasets used and/or analysed during the current study available from the corresponding author on reasonable request.
