# Supplementary material for: Therapy options for those affected by a long lie after a fall: a scoping review
Source: BMC Geriatr. 2022 Jul 15;22:582. doi: 10.1186/s12877-022-03258-2 (PMC9284880; doi:10.1186/s12877-022-03258-2)
Supplement: Supplementary file 1 — Additional file 1. [file 12877_2022_3258_MOESM1_ESM.docx]

Appendix I: Search string for PubMed

| Search string for PudMed |
| --- |
| (long lie* OR  inability to get up OR  immobil* OR  helpless OR  rhabdomyolys* OR  trauma)  AND  (fall* OR  drop* OR  accidental fall* [MH])  AND  (therap* OR  treatment OR  nurs* OR  care* OR  rehabilitation [MH]) |
